# Postoperative long-term results for the comparison of the symmetry of the upper lip during lip closure according to Millard and Pfeifer

**DOI:** 10.1186/s40902-018-0157-1

**Published:** 2018-08-01

**Authors:** Philipp Kauffmann, Robert Cordesmeyer, Giséle Awondzeko Fouellefack, Boris Schminke, Karl-Günther Wiese

**Affiliations:** Department of Oral and Maxillofacial Surgery, Georgia Augusta University, Robert-Koch-Strasse 40, 37099 Göttingen, Germany

**Keywords:** Lip cleft, Millard, Pfeifer, Cleft surgery, Lip symmetry

## Abstract

**Background:**

Clefts in newborns are associated with severe morphological and functional impairment. Especially the lip is of importance as if the treatment result is unsatisfactory, it can lead to psychological changes in the patient. Different operative procedures have been developed over the last decades. The aim of the presented study was the comparison of the surgical techniques according to Millard and Pfeifer regarding the temporal development of the postoperative symmetry of the lip height and mouth width.

**Methods:**

Digitized photographs of patients from the department of oral and maxillofacial surgery at the University of Göttingen were evaluated from 1979 to 1996. With a video analysis program, the lip height and mouth width were analyzed regarding the symmetry. We demonstrated the symmetry values over a period of 8 years in order to show the influence of growth on postoperative results.

**Results:**

The development of the vertical symmetry of the Philtrum and the lip vermillion on the cleft side in comparison to the healthy side behaves differently depending on Pfeifer and Millard. The lip height of the cleft lip was shorter in both techniques than on the healthy side, but Pfeifer’s difference was significantly more pronounced. The lip vermillion height on the cleft side was slightly shorter in the Millard group and markedly larger in the Pfeifer group. Both techniques can achieve good symmetry results for the vertical dimension of the lip. According to Pfeifer, the development of the horizontal dimension on the cleft side is bigger within the first 4 years than on the healthy side; according to the Millard technique, the horizontal development is smaller. These differences are greater within the first 6 years and approach between the 6th and 8th year.

**Conclusions:**

The Millard technique demonstrates better results concerning the philtrum and vermillion symmetry during growth within the first 6 years. Over the whole study period, growth corrects the philtrum and vermillion symmetry within the Pfeifer group.

## Background

**Fig. 1:**
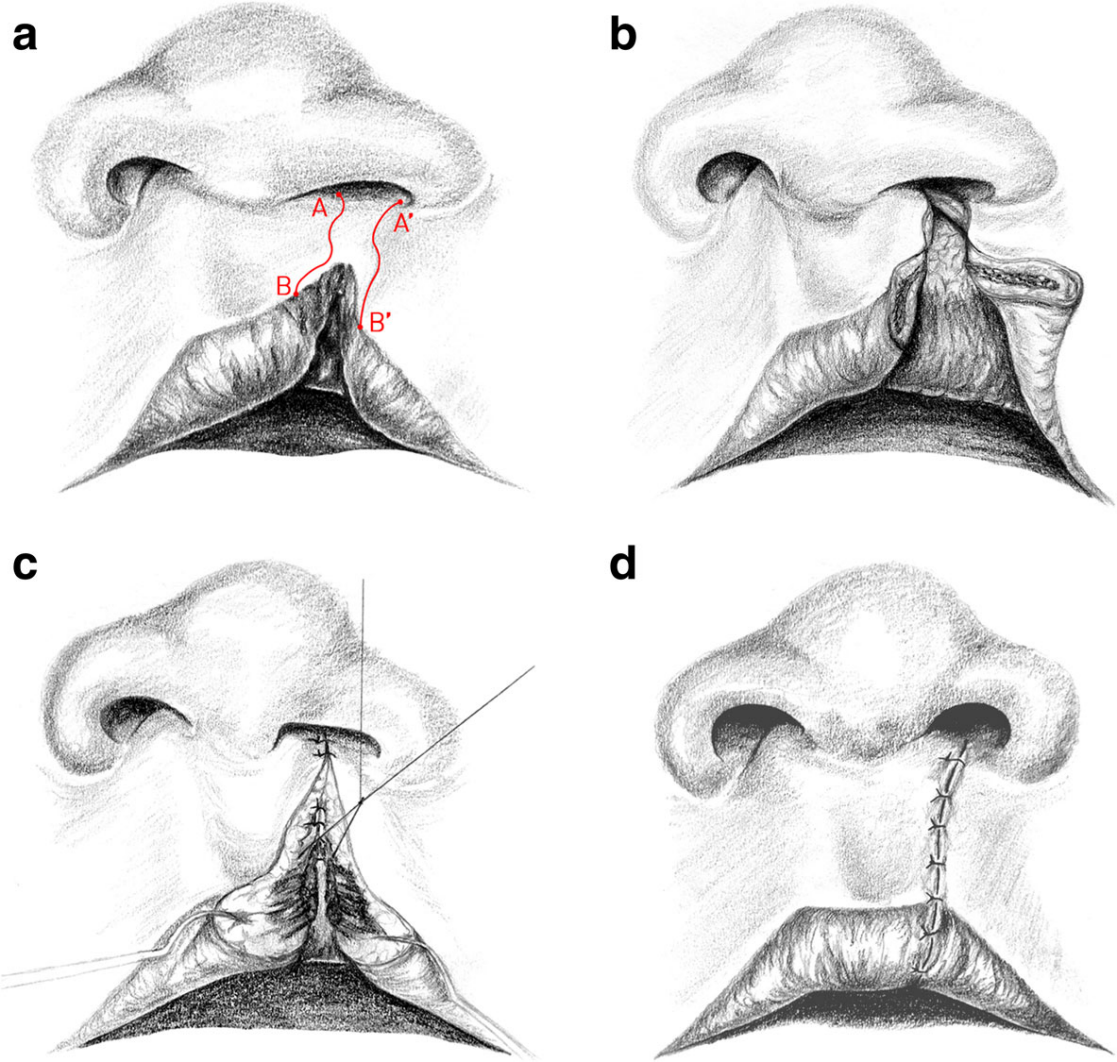
Operation method according to Pfeifer. **a** The planned red wave cuts A to B and A′ to B′ have the same length on both sides when they get stretched. **b** The orbicularis oris muscle is mobilzed from his wrong insertion on both sides subcutan and submucosally. **c** Reconstruction of the orbicularis oris muscle. **d** Result after wound closure (A to A′ and B to B′)

Lip and palate clefts, isolated lip, and isolated cleft of the palate have a prevalence of approximately 1/500 births and are a common cause of early morbidity in western countries [1]. Clefts in newborns are associated with severe morphological and functional impairment, which are accompanied by psychological damage in the course of child development [2]. The rehabilitation of these patients is of great importance since facial expressions and language are essential in interpersonal communication. For this reason, morphological damage should be removed by reconstructive-surgical procedures, deleting the anatomical, functional, esthetic, and psychological consequences. The lip is of importance because if the treatment result is unsatisfactory, it can, as mentioned before, lead to psychological changes in the patient. Until today, many surgical techniques have been developed for the closure of one-sided and two-sided lip clefts. The first techniques were described by Yperman from 1295 to 1350; strictly straight-line incisions were used without regarding the anatomical and functional structures [3]. Le Mesurier founded the era of angled cutting in 1949, followed by Tenisson in 1952 and Millard in 1955, who himself modified his technique [4–7]. The so-called wave-cutting method was first applied by Pfeifer in 1967 and uses the elasticity of the skin [8]. Using the Pfeifer method, a wrong insertion of the orbicularis oris muscle is mobilized subcutan and submucosally and united with the opposite side. The wave cuts are planned in such a way that on both sides the same length is created when the skin is being stretched. Afterwards both sides are sewn together (Fig. 1a–d). The Millard technique is widely used throughout the world, while the Pfeifer method has been preferred in the German-speaking world since the 1970s. There are currently no results on the vertical height and horizontal width of the lip symmetry of both methods. It was therefore the aim of this study to investigate these parameters postoperatively, while considering the time-dependent course.

## Methods

**Fig. 2:**
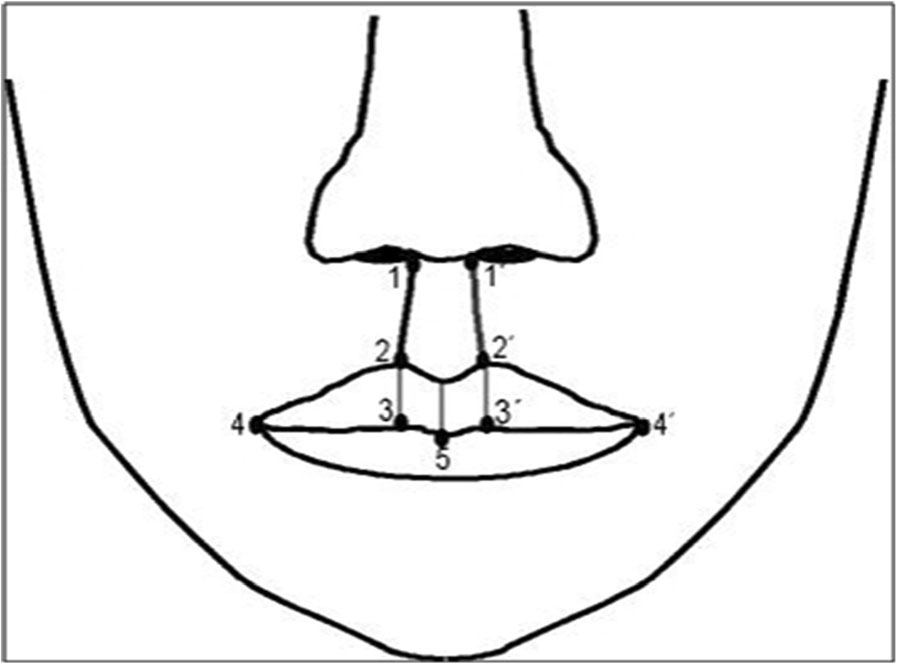
Reference points of the measured structures

The study was in accordance with the ethical standards of the University of Göttingen (No.: 35/5/18). The study is based on the analysis of digitized slide photographs of patients from the Department of Oral and Maxillofacial Surgery at the University of Göttingen, which were operated in this department from 1979 to 1996. During this period, an average of 40–50 primary cleft operations per year was performed. For the study, however, only patients with unilateral clefts with at least two postoperative “en face” control photographs were included in the evaluation. Furthermore, to show the effect of growth on the postoperative results, it was important that no surgical correction in the lip and nose area was performed during the follow up period. The mouth-nose area had to be clearly and completely represented on all images. High-resolution digital images were generated and measured using the video analysis program (SigmaScanPro, SPSS Science Inc., Chicago, USA). Figure 2 shows the reference points of the measured structures. The following distances were measured:

- Philtrum length of the healthy and the cleft side (1–2; 1′–2′).
- Lip vermillion length of the healthy and the cleft side (2–3, 2′–3′)
- Lip width of the healthy and the cleft side measured from the mouth angle to the lower lip vermillion middle (4–5; 4′–5).

The study includes only patients (*n* = 35) which were continuously observed over a period of 8 years post operatively. Table 1 gives an overview of the composition of the examination groups. In total, 80 photographs were evaluated and were assigned to postoperative time intervals. Months on the time axis were converted into a decimal year value. Three months therefore corresponds to 0.25 years. Each measurement was carried out by two investigators, and in the case of strong deviations of the absolute values in millimeters (> 10%), by a third investigator. From the measured values of the photographs, mean values were calculated. To eliminate error in range due to different magnifications we calculated a dimensionless quotient (Q) for the corresponding distances from these mean values, the cleft side (CS) being divided by the healthy side (HS) Q = CS/HS. This allows us to compare the values with one another independently of a photo magnification. A symmetry quotient of 1 represents perfect symmetry. A value > 1 means the cleft side is larger than the healthy side, and a value < 1 means the cleft side is smaller than the healthy side.

**Table 1:** Cleft Manifestation and operation procedure of the study Patients

OP method	Incomplete lip cleft	Complete lip cleft	Lip and alveolar cleft	Lip, alveolar, and palate cleft	Male	Female	Total
Millard	4	4	2	6	9	7	16
Pfeifer	4	5	2	8	16	3	19
Total	8	9	4	14	25	10	35

Overview of the composition of the examination groups

The means of the normally distributed measurement values of the Millard (*n* = 36) and Pfeifer (*n* = 44) group were compared to each other for significance testing. Subsequently, a *t* test for the comparison of the two unrelated groups was used at a significance level of *α* ≤ 0.05.

## Results

All patients at the time of surgery were at the age of 5–7 months (mean age 5.8 months). A total of 80 postoperative photographs were measured: 36 for the Millard Group and 44 for the Pfeifer Group.

**Fig. 3:**
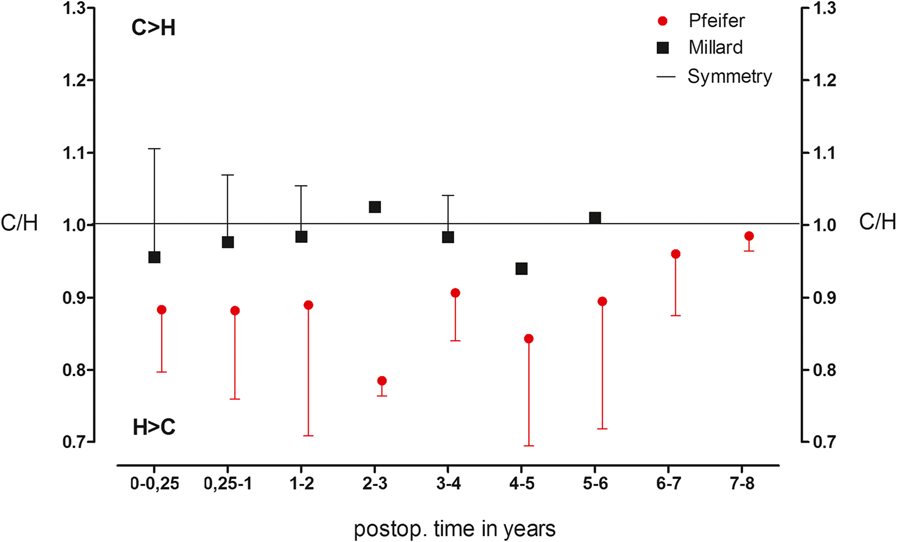
Relation of Philtrum length. Symmetry quotient for the Philtrum length over the entire follow-up period in the Millard group (*n* = 36) compared to the Pfeifer group (*n* = 44) (*p* < 0.001). Presented as the means ± standard deviation (SD)

Up to the fourth postoperative year, the Millard technique almost achieved a perfect symmetry with small deviations (2–5%) for the Philtrum length. In the Pfeifer group, the deviations were larger (10–22%), which is reflected in a shorter Philtrum on the cleft side. By comparing the means of the total time depended values, a significantly better symmetry quotient (*p* < 0.001) for the Philtrum length was found in the Millard group (*n* = 36) with 0.97 to 0.88 in the Pfeifer group (*n* = 44) (Fig. 3).

**Fig. 4:**
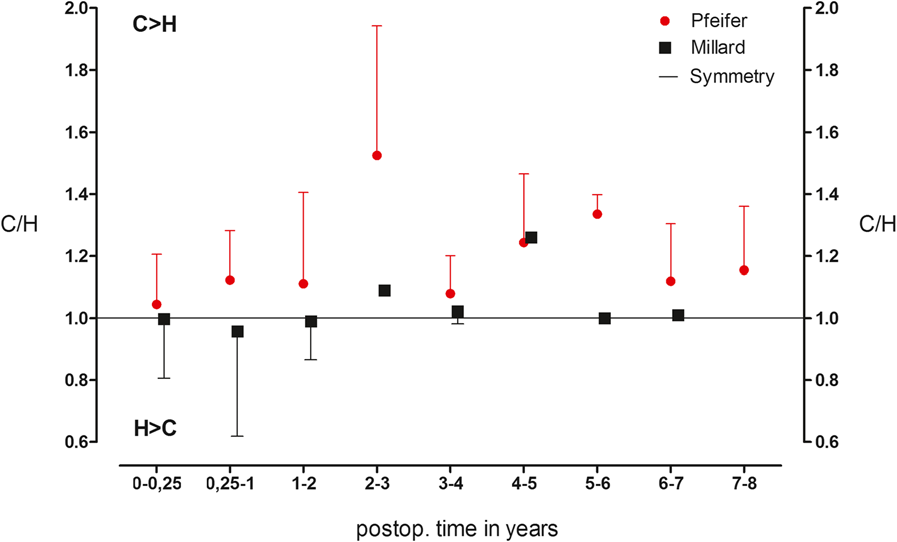
Relation of vermillion length. Symmetry quotient of the lip vermillion length over the entire follow-up period in the Millard group (*n* = 36) compared to the Pfeifer group (*n* = 44) (*p* = 0.003). Presented as the means ± standard deviation (SD)

The mean of the symmetry quotient of the lip vermillion length over the entire follow-up period was also significantly different from 0.99 in the Millard group (*n* = 36) and 1.43 in the Pfeifer group (*n* = 44), *p* = 0.003 (Fig. 4)**.** In the Pfeifer group, values above 1 showed a significantly longer lip vermillion on the cleft side.

**Fig. 5:**
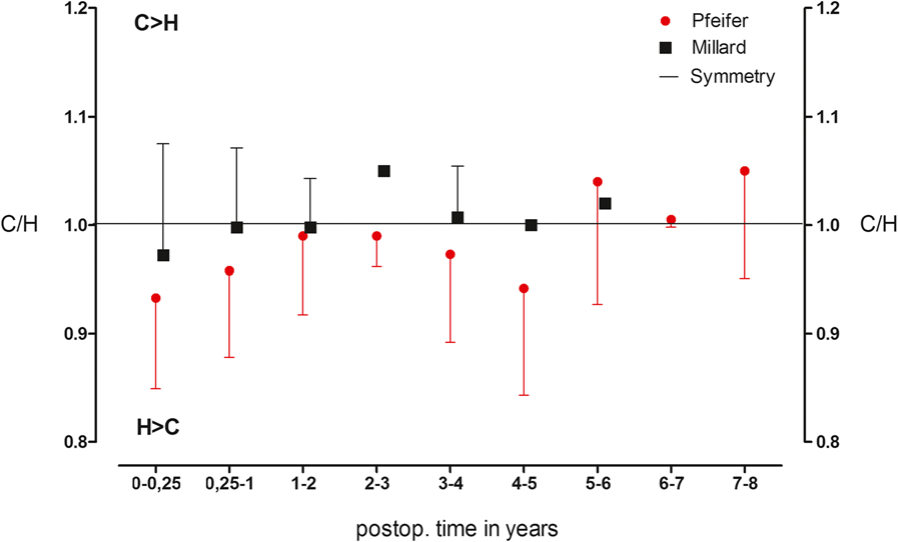
Relation of total upper lip height. Symmetry quotient of the total lip height in the Millard group (*n* = 36) compared to the Pfeifer group (*n* = 44) (*p* = 0.208). Presented as the means ± standard deviation (SD)

The total lip height, however, did not differ between the two groups. For the Millard group, the mean of the quotient is 0.98 (*n* = 36), and for the Pfeifer group, it is 0.96 (*n* = 44). (*p* = 0.208). Here, an almost perfect symmetry is achieved in both techniques (Fig. 5).

**Fig. 6:**
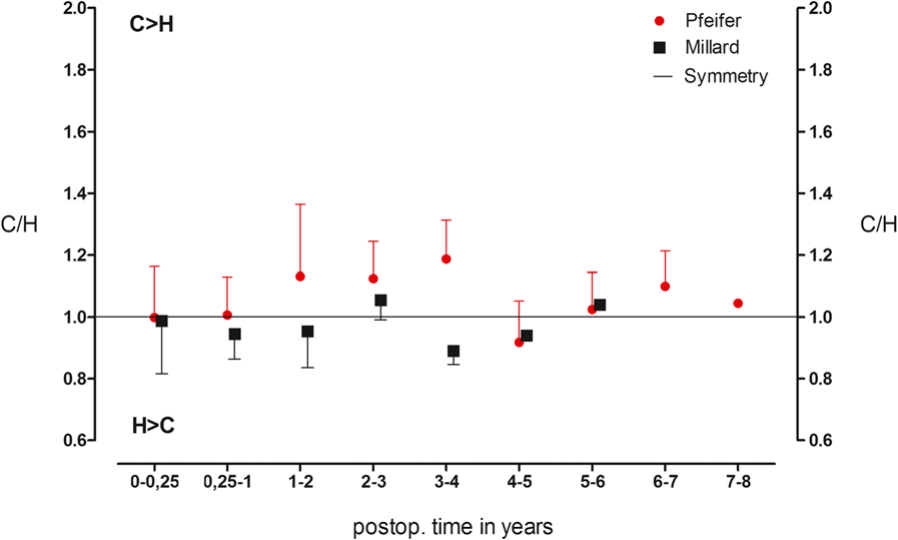
Relation of mouth width. Symmetry quotient of mouth width in the Millard group (*n* = 36) compared to the Pfeifer group (*n* = 44) (*p* = 0.156). Presented as the means ± standard deviation (SD)

If the ratio of the mouth width is considered, different and common patterns are shown for both techniques (Fig. 6).

The development of the horizontal dimension of the cleft side is, according to Pfeifer, generally larger up to the fourth year than on the healthy side. The Millard technique showed the reverse. From the age of four on both groups, however, it approached the value 1. With a *p* value of 0.156, the differences were not significant.

**Fig. 7:**
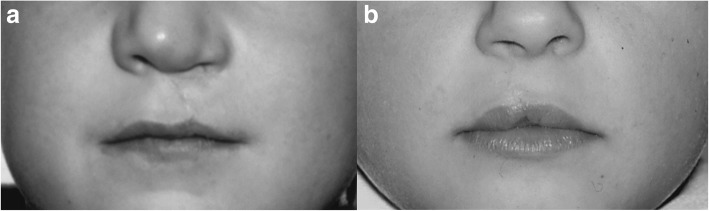
Postoperative results. **a** Clinical result 6 years postoperative after lip closure according to Millard. **b** Clinical result 6 years postoperative after lip closure according to Pfeifer

Good results can be achieved with both techniques (Fig. 7).

## Discussion

At birth, the lip is one of the most developed organs of the human body. The lip terminates its intensive development at preschool age [9]. At the age of 2 to 9 years, the lip growth shows a difference between girls and boys. The lip is larger during this age range among boys than in girls [10]. Lee [11] and Randal [12] criticized surveys of photos: since infants do not keep quiet, photographs are not standardized, and different enlargements can occur. Therefore, we avoided to use absolute values for the measurements. In his study, Lee carried out the measurements directly on the patient. This is also problematic since small children cannot keep quiet, the skin is soft and elastic and the measurements are difficult to reproduce. To circumvent the retrospective aspect of the no longer exactly determinable factor growth, only relative values were considered in this study. Therefore, errors of different imaging scales in photographs are eliminated because the values are simply normalized.

The final result of the cleft closure is essentially determined by the OP technique, the operator and the postoperative growth in the former cleft region. The wave-cutting technique, which had already proved itself in correctional procedures, was adopted as a primary treatment because it is more tissue-friendly and more flexible than the earlier methods. In addition, it uses the elasticity of the skin at the edge of the lip for lengthening the lips evenly [8]. Of importance is the liberation of the musculature from an unphysiological attachment and the exact union of the circular muscle [13]. This technique has a wide range of variations and allows individual consideration of the shape of the lip stumps [14]. Another great advantage lies in the precise measurement possibility [15].

Millard’s rotation-advancement principle is used most frequently in its modifications worldwide. Millard himself called it “cut as you go” [16]. It is very flexible and allows modifications during the cutting and the lip closure [17]. The technique is tissue-saving and allows a complete mobilization of the lateral nasal ala and its placement in the appropriate position so the symmetry of the nostrils and the nasal floor can be restored at the same time. Because of the flow of the advancement flap, not only a balanced height compensation of the lip is possible but also a volume adjustment of the cleft side [15].

Our results on the lip Philtrum in the Millard group agree with the statement by Millard that shortly after the operation, the cleft side was shorter than the healthy side, but compensation took place over time [6]. He also postulated that, in the case of wide clefts, a contraction of the cleft could be postoperative in the first 3 months. However, if the technique was applied properly, the result improved without any additional surgery after 6 months and showed almost perfect symmetry after 1 year. Investigations by Becker even showed a lengthening of the lips on the cleft side [18].

Lee [11] carried out measurements on the lip immediately after the operation and determined that incomplete clefts had a symmetry between the cleft and the healthy side, while complete lip clefts were shorter on the cleft side than on the healthy side. No improvement was noted a year postoperatively. Le Mesuier, Mulliken and Martinez-Perez, and Saunders et al. [4, 19, 20] also reported a shortened Philtrum on the cleft side according to the Millard technique. Millard acknowledged that the asymmetry was in some cases caused by the defect at the maxilla and recommended a lip adhesion operation, which already had been recommended by other authors [21–23], as well as a preoperative orthodontic treatment [24]. Additionally, in Göttingen, the patients with lip-to-palate clefts were provided with the Latham plate before lip closure to position the jaw segments. This could explain why most of the patients in the present study had a symmetrical Philtrum until the fourth postoperative year. As clearly confirmed by our study, the symmetry course shows a clearly time-dependent behavior.

In the Pfeifer group, the Philtrum is briefly shorter after the operation and in the further examination course on the cleft side than on the healthy side. Although this technique is very common in the German-speaking world, it is not often reported in the literature. Maerker and Bull [14] found that the red and white border was symmetrical in 58 out of 83 examined children, whereas in 25 examined children, the cleft lip Philtrum showed a shortening and thus a distortion of the lip vermillion with step formation. Bitter [15] also noted a shortening of the lip Philtrum.

Regarding the vermillion length, we found contradictory statements. Mulliken and Martinez-Perez [19] found a very full vermillion in the patients who were operated with the Millard technique, whereas Noordhoff describes a deficit [25]. These differences can also be explained with the different temporal course.

Märker and Bull found a very full upper lip in patients who had been operated with the Pfeifer technique [14].

In the present study, the total lip height is not significantly different in both groups. However, in the Pfeifer group, the deficiency of the Philtrum is compensated by a too plumb vermillion. This can be accompanied by esthetic limitations. For this reason, Pfeifer recommended that in case of an esthetic deficiency, a correction should be completed before the first day of school [8].

Over the whole study period, the two groups did not differ in mouth width, but the development of the horizontal dimension of the cleft side is, according to Pfeifer, until the fourth year, bigger than the healthy side; in the Millard group, it is the other way around. Lewis [26] also observed a narrowing of the lip width in the Millard group postoperatively. According to our study, Märker and Bull (1982) observed a widening in the first postoperative years [14].

## Conclusions

The continuous presentation of the postoperative results in the same patients show that the vertical and horizontal dimensions of the upper lip undergo dynamic changes during the observation period in both surgical procedures. These differences demonstrate how the two operation procedures effect the lip growth postoperatively. The Millard technique demonstrates better results concerning the philtrum and vermillion symmetry during growth within the first 6 years. Over the whole study period, growth corrects the philtrum and vermillion symmetry within the Pfeifer group. Therefore, it seems to be advisable not to perform corrections of the lip, in case of absence of complications, before the 6th to 7th year of life.

## Abbreviations

CS: The cleft side
HS: Healthy side
Q: Dimensionless quotient for the corresponding distances from these mean values
SD: Standard deviation
